# Short-term efficacy of stenting as a rescue therapy for acute atherosclerotic occlusion in anterior cerebral circulation

**DOI:** 10.3389/fneur.2023.1238998

**Published:** 2023-11-01

**Authors:** Jianyi Wang, Suhang Shang, Wanghuan Dun, Chen Chen, Fan Gao, Jia Yu, Jianfeng Han, Fude Liu

**Affiliations:** ^1^Department of Neurology, The First Affiliated Hospital of Xi'an Jiaotong University, Xi'an, Shanxi, China; ^2^Department of Rehabilitation, The First Affiliated Hospital of Xi'an Jiaotong University, Xi'an, Shanxi, China; ^3^Clinical Research Center, The First Affiliated Hospital of Xi'an Jiaotong University, Xi'an, Shanxi, China

**Keywords:** atherosclerosis, acute ischemic stroke, endovascular therapy (EVT), Neuroform EZ stent, efficacy, rescue therapy

## Abstract

**Purpose:**

The study aimed to explore the efficacy and safety of the Neuroform EZ stent in treating acute anterior circulation large artery occlusion.

**Methods:**

The clinical data of 42 consecutive patients with acute anterior circulation large atherosclerotic occlusion who were treated with the Neuroform EZ stent from January 2018 to August 2019 in our stroke care center, including baseline characteristics, images, therapeutic condition, and follow-up data were retrospectively analyzed.

**Results:**

There were 42 mechanical thrombectomy (MT) failure cases of intracranial atherosclerotic stenosis with rescue Neuroform EZ stent implantation, of which 78.6% (33/42) had a good prognosis and 88.1% (37/42) showed no re-stenosis at follow-up. The average time from puncture to recanalization is 79.50 ± 14.19 min. The successful rate of intraoperative stent release is 97.6%, while there is one case of stent displacement, three cases of thrombus escape, and six cases of hemorrhage.

**Conclusion:**

Rescue therapy of the Neuroform EZ stent for acute anterior circulation large atherosclerotic occlusion can archive good short-term imaging and clinical results, while long-term follow-up is still needed to verify.

## Introduction

Recently, multiple landmark trials (MR CLEAN, ESCAPE, SWIFT PRIME, REVASCAT, and EXTEND IA) indicated that endovascular therapy significantly benefits patients exhibited with acute ischemic stroke caused by large artery occlusion in the anterior circulation (LVO) (1–5). In particular, the DEFUSE3 (endovascular treatment following imaging evaluation for ischemic stroke 3, DEFUSE 3) and DAWN (DWI or CTP assessment with clinical mismatch in the triage of wake up and late presenting strokes undergoing neuro-intervention with Trevo, DAWN) studies expanded the indication of EVT therapy beyond time windows into the era of tissue window assessment (6, 7), which further expanded the number of ALVO stroke patients (8). The majority of the patients included in those clinical trials were due to embolic LVO instead of intracranial arteriosclerotic stenosis (ICAS), while ICAS is often more common in Asian, Hispanic, and African populations. It is clear which type of EVT tools are used for embolic ALVO, while the evidence regarding ICAS-related ALVO is insufficient.

Recanalization for ICAS-related ALVO patients did not process *in-situ* stenosis. Thus, it usually achieves temporal blood flow and aggressively triggers platelet aggregation leading to artery re-occlusion (9). Endovascular Therapy for Acute Ischemic Stroke Trial (EAST) indicated that the proportion of ICAS-caused ALVO patients requiring rescue therapy after successful mechanical thrombectomy (MT) recanalization was as high as 63.8% (10). Previous research study has shown that the Neuroform EZ stent achieves a good image and clinical results for symptomatic ICAS patients (11). Emergency intracranial stenting is a vital rescue method after failed MT (12), yet the efficacy of stent and the optimal type of stent to quickly reconstruct blood flow is less reported. This prospective study aimed to investigate the efficacy and safety of the Neuroform EZ stent as an emergency rescue therapy after failed MT in ICAS-related ALVO patients.

## Methods

### Study population

The clinical data of 42 consecutive patients with ALVO in the anterior circulation who were admitted to the First Affiliated Hospital of Xi'an Jiaotong University and underwent rescue therapy using Neuroform EZ stenting were analyzed.

#### Inclusion criteria

(1) Patient ≥ 18 years of age, (2) ALVO due to anterior circulation (ICA, MCA-M1, and/or M2 segment) by magnetic resonance angiography (MRA) or digital subtraction angiography (DSA), (3) CT scan within 6 h from symptom onset or 6–24 h from symptom onset and meet DAWN criteria, (4) baseline modified Rankin scale (mRS) score of ≤ 2, (5) Alberta stroke program early CT score (ASPECTS) ≥ 6, (6) baseline National Institutes of Health stroke scale (NIHSS) ≥ 6, and (7) ALVO due to ICAS confirmed by DSA.

#### Exclusion criteria

(1) Intracranial hemorrhage history or bleeding diathesis, (2) massive cerebral infarction on CT or MRI (infarct volume ≥ 70% or infarct volume > 1/3 MCA territory, ASPECTS < 6 from CT/DWI), (3) ALVO caused by non-atherosclerotic etiology, (4) tandem lesion (postal intracranial artery occlusion combining with 100% or no <90% occlusion of extracranial carotid artery) (13), (5) pre-operate platelet count <50 × 10^9^/L, (6) random blood sugar < 2.78 or >22.2 mmol/L, (7) uncontrolled hypertension (systolic blood pressure, SBP > 185 mmHg, or diastolic blood pressure, DBP > 110 mmHg), (8) severe cardiac, pulmonary, or renal diseases, and (9) life expectancy <90 days. The institutional review boards of all participating hospitals approved this study and waived the requirement for informed consent for study inclusion based on the retrospective study design.

### Clinical data collection

Patient's clinical data were collected, including demographics, stroke risk factors, baseline blood test, baseline BP level, territory artery, preprocedural IV rt-PA, type of anesthesia, baseline ASPECTS score, and time from puncture to recanalization.

### Procedure description

Patients who met the criteria within 4.5 h of symptom onset were treated with I.V. rt-PA before MT. Under general anesthesia or conscious sedation, depending on the patient's clinical status, a transfemoral approach with an 8F sheath (Terumo, Terumo Medical, Tokyo, Japan) was used to access the occlusion lesion and compensation of collateral circulation. Then, an 8F guiding catheter and 6F (125 cm length) supporting catheter system were placed to provide stable surgical support. The Solitaire stent-retriever thrombectomy device (Covidien, Irvine, California, USA) was attempted first to restore the forward re-flow. If the reflow cannot be maintained for 15 min or the artery occludes again, the operator rapidly switches to an expansion balloon to slowly and evenly dilate the lesion site. With observing apparent elastic retraction or the local dissection-like lesion, the head of the XT-27 microcatheter (Stryker) is positioned at the distal end of the stenosis through the long exchange guidewire. Subsequently, the Neuroform EZ stent (Stryker) was positioned with precision to cover the lesion completely, which requires the stent's diameter to be slightly larger than the inner diameter of the blood vessel, the length of the stent to exceed the narrowing or occlusion segment by at least 3 mm at both ends, and the mark points to be well-opened and adhered to the wall at both ends of the bracket. The radiological outcome was measured using the mTICI scale with successful recanalization defined as a mTICI score of 2b or 3 (14). A successful angioplasty was defined as coverage of the targeted stenosis lesion by the stent and residual stenosis of no more than 50%. The degree of all artery stenosis is measured according to the Warfarin-Aspirin Symptomatic Intracranial Disease (WASID) study (15).

### Periprocedural anti-thrombotic treatment

In general, the strategy of anti-thrombotic therapy is highly individualized. Only patients who received direct MT were given systematic heparinization. After loading dosage of glycoprotein IIb/IIIa inhibitor (tirofiban) was administered intravenously, maintenance dosage was administered for 24 h when immediate post-surgical CT ruled out interracial hemorrhage (ICH). After overlapping with oral loading dosage dual anti-platelet (aspirin 300 mg and clopidogrel 300 mg) medicine for 6 h, intravenous tirofiban was discontinued after a negative CT scan 24 h post-procedure. A routine regimen of oral dual anti-platelet (aspirin 100 mg, according to CYP2C19 genetic guidance to choose clopidogrel 75 mg or Tegrilol 90 mg Bid) was taken for at least 3 months, followed by lifelong single antiplatelet therapy.

### Clinical and imaging follow-up

Primary efficacy endpoint: good outcome rate at 90 d post-procedure (mRS ≤ 2). Other endpoints include success rate of recanalization, stent release and angioplasty, incidence of intraoperative thrombus escape, vascular perforation bleeding rate, symptomatic intracranial hemorrhage (sICH) 24 h post-procedure, in-stent restenosis rate within 1 week, and all-cause mortality within 24 h and 90 days. The sICH 24 post-procedure is defined as a new cranial hemorrhage reviewed using a CT scan, which causes neurological deterioration (NIHSS increase of more than 4 points) or death within 24 h after surgery (16). Thrombus escape was defined as angiography indicating the appearance of a new embolism in a previously uninvolved watershed or distal to the targeted artery during MT (17). At the 6-month follow-up, computed tomographic angiography (CTA) was otherwise done for patients who refused to have DSA. In-stent re-stenosis is when the diameter of the stent or adjacent 5 mm to the stent is narrowed by over 50% (18).

### Statistics analysis

Analyses were conducted with SPSS V20.0 (IBM Corporation, Armonk, New York, USA). Count data were expressed as rate (%). Measurement data conformed to a normal distribution were expressed as ($\bar{x\;}\pm$ s) and otherwise expressed as median (P25–P75). A *p*-value of < 0.05 was considered to be statistically significant.

## Results

Our study enrolled 42 ICAS-related ALVO patients, all of whom received stent implantation, followed by stent retriever and balloon angioplasty. There were 30 male (74.1%) and 12 female (25.9%) patients, with an average age of 64.24 ± 11.35 years. Additionally, there were 12 patients with hypertension, 3 patients with atrial fibrillation, 21 patients with diabetes, and 4 patients with a history of ischemic stroke or TIA. The median NIHSS score was 10 (7–13) and the median ASPECTS score was 8 (7–9). Among the 42 patients, 28 had excellent collateral compensation, 8 (19.1%) were located in the C5–C7 segment of the internal carotid artery, 17 (40.5%) were located in the proximal 1/2 of the M1 segment of the middle cerebral artery, 14 (33.3%) were located in the distal 1/2 of the M1 segment of the middle cerebral artery, and 3 (7.1%) were located in the M2 segment of the middle cerebral artery (results seen in Table 1).

**Table 1:** Baseline characteristics.

Age, y; mean ± SD (*N*)	64.24 ± 11.35 (65)
Men (%)	30 (71.4%)
IV rt-PA before MT/(%)	4 (9.5%)
Baseline NHISS score; median(1QR)	10 (7, 13)
ASPECTS score; median(1QR)	8 (7,9)
Good collateral compensation (%)	28 (66.7%)
Time from puncture to procedure closure, median ± SD/min	79.50± 14.19 min
**Risk factors**	
Hypertension (%)	12 (28.6%)
Atrial fibrillation (%)	3 (7.1%)
Diabetes mellitus (%)	21 (50%)
TIA or stroke (%)	4 (9.5%)
**Occlusion site**	
C5–C7 of ICA	8 (19.1%)
Proximal M1 segment of the MCA	17 (40.5%)
Distal M1 segment of the MCA	14 (33.3%)
M2 segment of MCA	3 (7.1%)

Values are presented as number, n (percentage, %) unless indicated otherwise. SD indicates standardized difference; TIA, transient ischemic attack, NIHSS, National Institutes of Health Stroke Scale, IQR, interquartile range, ASPECTS, Alberta Stroke Program Early CT Score, ICA, internal carotid artery, MCA, middle carotid artery, IV rt-PA, intravenous recombinant tissue-type plasminogen activator, MT, mechanical thrombectomy.

Among these patients, 4 (9.5%) received IV rt-PA before MT, and 38 received direct MT. Of all patients who recanalized with mTICI > 2C, 31 (73.8%) achieved recanalization with first retrieve, and 38 (90.5%) recanalized with grade 3 mTICI. The overall rate of residual stenosis was <50%, among those 33 cases exhibiting a rate of no more than 30% and 9 cases exhibiting a rate of 30–50%. The average time from a puncture-to-procedure closure is 79.50 ± 14.19 min. There are three cases of thrombus escape after Neuroform EZ stent implantation, while one case was recanalized at grade 3 mTICI after remedial thrombus aspiration, and the other two cases were recanalized at grade 2b mTICI following intra-arterial thrombolysis with Tirofiban. The targeted artery did not suffer any perforation, in-stent restenosis, or re-occlusion, but six patients (14.3%) did experience hemorrhage transformation, which was reviewed by dynamic CT immediately and for a period of 24 h post-operation. Among the patients enrolled, five patients experienced parenchymal hemorrhage and one patient experienced subarachnoid hemorrhage (SAH) (results details seen in Table 2).

**Table 2:** Radiologic and clinical outcomes after endovascular treatment.

**Efficacy outcomes**	
Final successful reperfusion, *n* (%)	42 (100%)
Successful reperfusion after first attempt of thrombectomy, *n* (%)	31 (73.8%)
**mTICI score post procedure**, ***n*** **(%)**	
2b	4 (9.5%)
3	38 (90.5%)
**Degree of residual stenosis**, ***n*** **(%)**	
≤ 30%	33 (78.6%)
30%~50%	9 (21.4%)
Time from puncture to procedure closure, median ± SD/min	79.50 ± 14.19
Thrombus escape, *n* (%)	3
**Safety outcomes**	
Any hemorrhagic transformation, *n* (%)	6
sICH	5
Ipsilateral basal ganglia hemorrhage	2
Lethal bleeding	1
Parenchymal hematoma remote from infarcted brain tissue	2
SAH	1
Stent displacement, *n* (%)	1
90 d mRS no more than 2, *n* (%)	33 (78.6%)
DSA or CTA completion at follow-up	37 (88.1%)
Improvement of residual stenosis on follow-up image	12 (32.4%)

Values are presented as number, n (percentage, %) unless indicated otherwise. SD indicates standardized difference; mRS, modified Rankin score; sICH: symptomatic intracranial hemorrhage; SAH, subarachnoid hemorrhage; DSA, Digital Subtraction angiography; CTA, computed tomographic angiography.

We followed all 42 patients and did not find any TIA, stroke, or death during follow-up, and 78.6% of patients have a good prognosis with mRS of no more than 2. In total, 37 cases completed DSA or CTA follow-up at a time of 3–6 months, and none of them revealed any evidence of in-stent stenosis. Additionally, 12 cases (32.4%) showed improved residual stenosis compared to the immediate post-operative time (results seen in Table 2).

### Typical cases

#### Case 1

**Figure 1:**
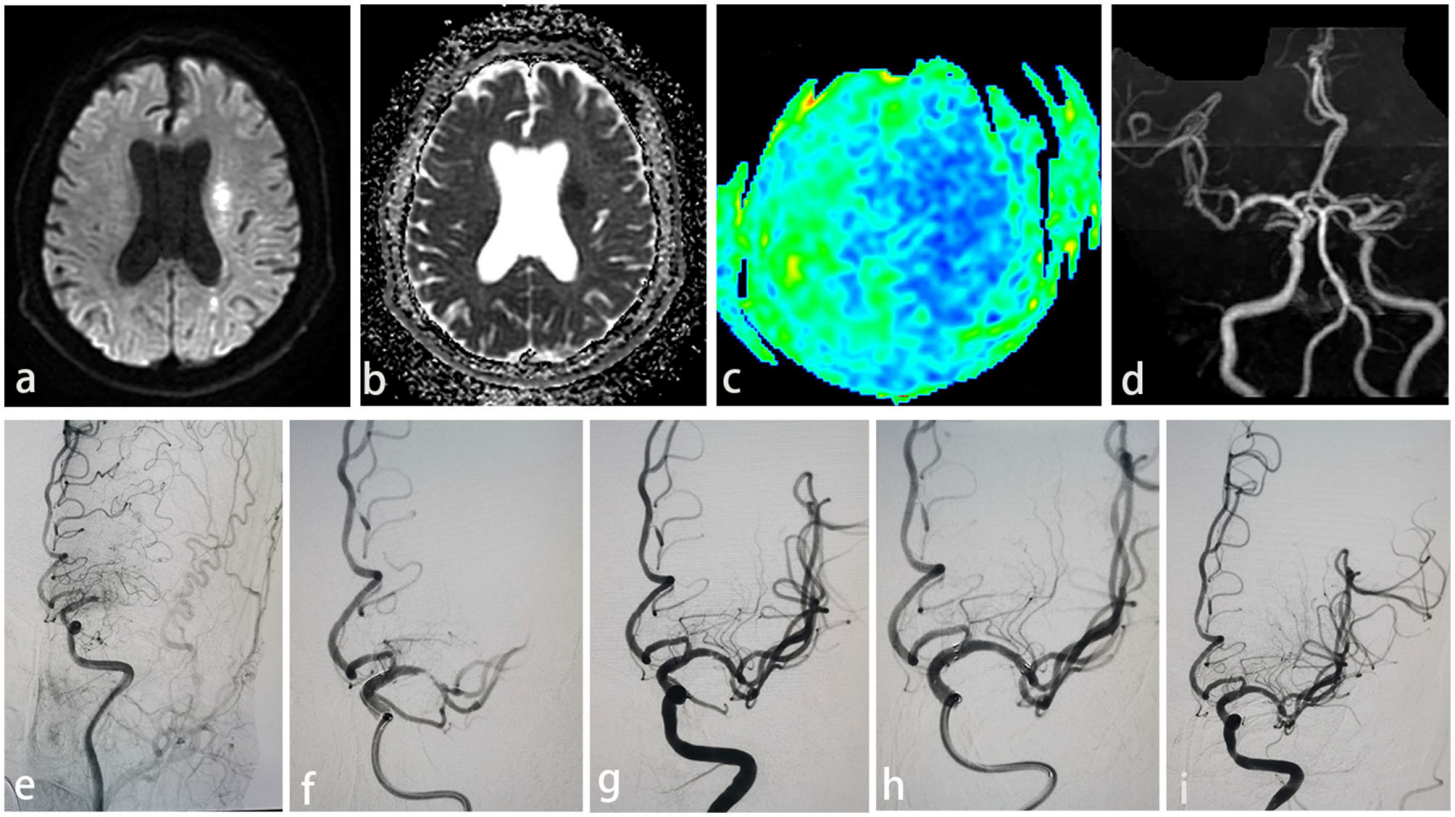
**(a)** Watershed infarction of left hemisphere in DWI. **(b)** Watershed infarction of left hemisphere in ADC. **(c)** Reduced perfusion of left hemisphere. **(d)** Occlusion of left MCA showing in MRA. **(e)** Acute left MCA occlusion in DSA. **(f)** Severe stenosis (>70%) with associated flow limitation after mechanical thrombectomy with a stent retriever. **(g)** Possible dissection after mechanical thrombectomy. **(h)** Deploy 3.0 mm × 15 mm Neuroform EZ stent via microcatheter XT-27. **(i)** Good patency and no in-stent stenosis in 6-months DSA follow-up.

A 56-year-old male presented with paroxysmal right limb weakness for 8 h, which then worsened for 3 h. Upon further neurological examination, we found somnolence, incomplete gaze to the left side, central facial palsy, complete motor aphasia, and grade 4 muscle strength in the right limb, with an NIHSS of 8. Multimodal MRI revealed a subcortical type of watershed infarction in the left hemisphere (Figures 1a, b) and reduced perfusion (Figure 1c) and occlusion of the M1 segment (Figure 1d). Under general anesthesia and with systematic heparization, DSA indicated occlusion of the left MCA (Figure 1e) and a microcatheter Headway21 guided by microwire Synchro (0.014", 200 cm, Stryker, USA) was carefully placed through the blockage site to the M2 segment within the supporting 8F guide catheter and 6F intermediate catheter. A Solitaire 4–20 mm stent was used to retrieve the embolus, and the subsequent angiography revealed severe stenosis in the left middle cerebral artery M1 segment (Figure 1f). With the balloon dilation showing potential dissection (Figure 1g), the stent was released smoothly and attached well, utilizing the microcatheter XT-27 with a 3.0 × 15 mm Neuroform EZ stent (Figure 1h). The repeated DSA after 6 months demonstrated satisfactory patency and no signs of re-stenosis in the stent (Figure 1i).

#### Case 2

**Figure 2:**
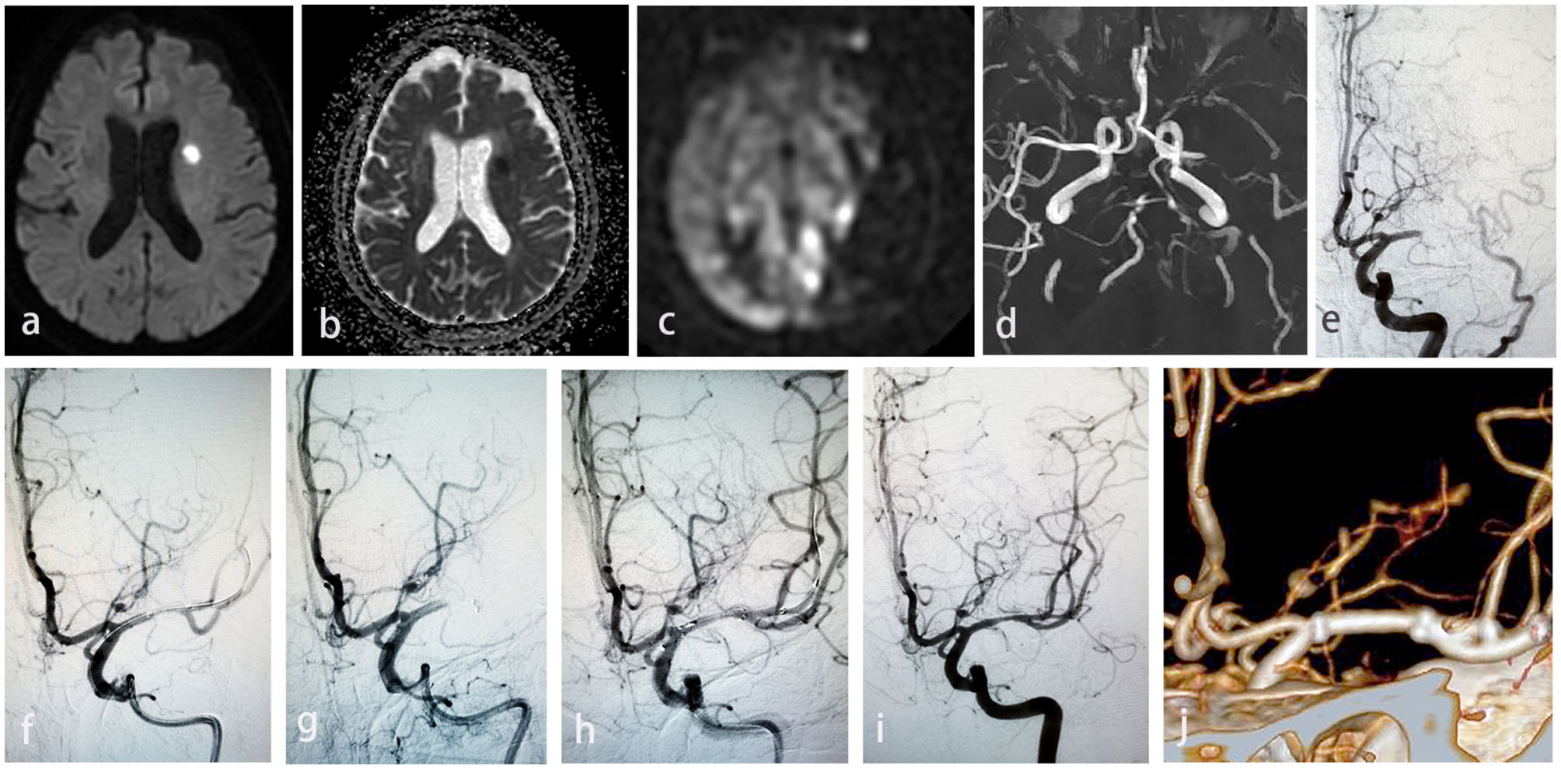
**(a)** Scattered infarction of left hemisphere in DWI. **(b)** Scattered infarction of left hemisphere in ADC. **(c)** Reduced perfusion in the left cerebral hemisphere. **(d)** Occlusion of left MCA in MRA. **(e)** Acute left MCA occlusion in DSA. **(f)** Severe stenosis in the left MCA after Solitaire stent retriever. **(g)** Re-occluded left MCA after misplaced stent caused by incomplete released tension of microcatheter XT-27. **(h)** Balloon dilation the occluded site of left MCA through micro guidewire. **(i)** 30% residual stenosis of target artery after stenting. **(j)** Good stent patency and relieved residual stenosis in CTA at 90 d.

A 77-year-old female presented with progressive left limb weakness and blurred speech for 8 h, and a physical examination revealed sensory aphasia, grade 2 muscle strength of the right limb, with an NIHSS of 6. MRI suggested scattered infarct foci in the left lobe (Figures 2a, b), reduced perfusion in the left cerebral hemisphere (Figure 2c), and occlusion of the left MCA in MRA (Figure 2d) and in DSA (Figure 2e). Under general anesthesia and with systematic heparization, microcatheter Headway21 guided by microwire Synchro (0.014", 200 cm, Stryker, USA) was carefully placed through the occlusion site to the M2 segment under the supporting of 8F guide catheter and 6F intermediate catheter, and then Solitaire 4–20 mm stent was used to retrieve the embolus and following angiography showed severe stenosis in the right middle cerebral artery M1 segment (Figure 2f). After balloon dilation, the stent was released via a microcatheter XT-27 with a 2.5 × 15 mm Neuroform EZ stent. As the incomplete released tension of microcatheter XT-27 resulted from the stent not being fully placed around the proximal end of the lesion, the following angiogram showed the artery was still occluded (Figure 2g), which drove us to place a micro guidewire into the distal stem of M2 segment and dilate the occluded site with a balloon. Then, a 2.5 × 20 mm Neuroform EZ stent was placed via XT-27 microcatheter with SL-10 (Stryker, USA) super-selected stent mesh into the upper stem (Figure 2h). Angiogram showed that the two stents were released in a “Y” shape, and the target artery had 30% residual stenosis (Figure 2i). The mRS is 1 score at 90 days, and CTA showed good stent patency (Figure 2j) and relieved residual stenosis.

## Discussion

ICAS is relatively more common in the Asian population, while the CICAS study [the prevalence and outcomes of symptomatic intracranial large artery stenoses and occlusions more in China: the Chinese intracranial atherosclerosis (CICAS study)] indicated that 46.6% of patients with stroke or TIA was caused by ICAS (19). *In-situ* thrombosis or embolization caused by plaque rupture in ICAS-related ALVO patients dramatically increases the risk of stroke mortality and disability rate and lowers the recanalization rate of intravenous rt-PA. EVT has become the first-line therapy to obtain reperfusion, eliminate potential stenosis, and prevent re-occlusion (20). The fundamental pathogenesis of ICAS-related ALVO is the intracranial artery's local *in-situ* severe stenosis. Mechanical thrombectomy can restore blood flow, while the platelet aggregation caused by the *in-situ* stenosis quickly leads to re-occlusion. Therefore, salvage therapy is necessary, and the identification of ICAS is of great significance. Medical history, including progressive, fluctuating course, a history of atherosclerosis, and imaging evidence, including negative SWI susceptibility vessel sign and CT middle cerebral artery high density, can aid in identifying ICAS-related ALVO. In addition, signs during the thrombectomy process, including the first passage effect of the microcatheter, residual stenosis after thrombectomy, the characteristics of the removed thrombus (the darker the color, the richer the red blood cells, and the more likely to be of cardiac origin), and the morphological change of the stent tip (no change in embolization and constriction of stent tip while passing through the stenosis due to lesion binding) were used to identify ICAS.

The optimal method of thrombectomy for ICAS-related ALVO is controversial. Recently, a meta-analysis showed that 84.1% of all patients who received MT-preferred stent retriever had an excellent recanalization rate of 88% (95% CI, 84–92%) and an improved outcome of 52% (95% CI, 47–56%) at follow-up (21). The stent retriever can rapidly achieve reflow as there is less thrombus burden in ICAS-related ALVO. Second, a stent retriever can determine the severity and length of the lesion site, characteristic of stenosis or occlusion, and further assist the operator in making remedial strategies. A large-caliber aspiration catheter is not the first choice because of the difficulty of operating through the tortuous or angioarchitecture anatomic pathways. As per our study, the relatively short Solitaire stent retriever (4–20 mm) was the preferred choice for ICAS-related ALVO, with a 73.8% recanalization rate of our first thrombectomy and a 100% successful vascular recanalization rate. We recommend early rescue angioplasty if the occlusion is still present after two failed stent retrievers.

Multiple studies indicate that stent thrombectomy alone cannot successfully achieve vascular recanalization, and ICAS-related ALVO often re-occludes after successful recanalization. The severity of ICAS lesions is one of the most critical factors of refractory occlusion (22, 23) as the more severe the stenosis, the higher the rate of re-occlusion. Multiple operations promote vulnerable plaque rupture and activate the inflammatory response (24, 25), which extensively increases the intimal injury and accelerates platelet aggregation (26). Moreover, multiple procedures increase device-related complications (vasospasm, arterial perforation, dissection, and device detachment/misplacement) (27, 28) and the incidence of new infarction or hemorrhage, which further aggravates neurological deficit and increases the length of stay in intensive care and stroke units (29). Therefore, there are several salvage treatments for re-occlusion after failed MT caused by ICAS-related ALVO, including the use of glycoprotein IIb/IIIa inhibitors, balloon dilatation, stent angioplasty, and combination therapy (23, 30–33).

The optimal rescue stent remediation remains unknown. On the one hand, the characteristic of ALVO-related plaque is more vulnerable and thrombogenic compared to severe stenosis plaque (34). On the other hand, the strong radical support force and rigid transportation system of Wingspan cause worrying perioperative complications in treating symptomatic intracranial stenosis. In the studies by Baek et al. and Wu et al. comparing patients treated with emergency angioplasty and/or stenting with patients who failed mechanical thrombectomy and received no further treatment, functional recovery was better in the rescue group than in the no-stent group (35, 36). Jia et al. analyzed that the effective recanalization rate of the Solitaire stent in patients with ICAS-related ALVO was 95.7%, and the 90-day good prognosis rate was 63.8% after rescue remedial measures (10). In our study, immediate intravenous tirofiban after MT was followed by slow balloon dilatation to achieve stable blood flow. When the elastic retraction of plaque was apparent or the local dissection was formed during the observation period, a self-expanding open-loop Neuroform EZ stent was used for angioplasty to prevent delayed re-occlusion. We summarize our results in several aspects. First, the *in-situ* stenosis of AIS-LVO caused by ICAS is mainly located in the M1 segment of the middle cerebral artery and the middle segment of the basilar artery, which have affluent perforators. The appropriate radial force of the Neuroform stent prevents elastic retraction of plaques and in-stent restenosis and also reduces the influence on the perforator artery by excessively reducing the snowplowing effect. Second, the open-loop stent has good adherence performance, which prevents stent thrombosis and occlusion and reduces the risk of bleeding due to traction by not altering the course of the blood vessel. Finally, the microcatheter delivery system has an excellent advantage for the tortuous artery or M2 segment because of its flexibility and ease of placement.

The good prognosis rate at day 90 in our study is 78.6%, and no restenosis in-stent or re-occlusion happened at CTA or DSA follow-up, which is within the range of the literature reported. There is one case of stent release displacement, and the reasons were that first, incomplete tension of the microcatheter during stent implantation results from inaccurate forward stent positioning. Second, the open-loop stent could not be retrieved and repositioned once released, which required a well-experienced operator. The main problem with permanent stent implantation is that antiplatelet aggregation can increase the occurrence of sICH. In this study, all patients were treated with a glycoprotein IIb/IIIa inhibitor and subsequently dual antiplatelet drug therapy for at least 3 months to resolve or prevent acute stent thrombosis. Therefore, there were six patients with ICH and two with sICH, but within the range of previous studies.

Our study demonstrates that it is safe and effective for the Neuroform EZ stent in treating ICAS-related ALVO, while some limitations are listed below. Our study is a single-center retrospective study with a small size and no control group. Second, there are varying degrees of deselection bias because the stent selection and lesion identification were based on the operators' experience. Moreover, last but not least, the follow-up period is too short, and long-term clinical and imaging are needed.

## Data availability statement

The original contributions presented in the study are included in the article/supplementary material, further inquiries can be directed to the corresponding author.

## Ethics statement

The studies involving humans were approved by Ethics Committee of the First Affiliated Hospital of Xi'an Jiaotong University. The studies were conducted in accordance with the local legislation and institutional requirements. Written informed consent for participation was not required from the participants or the participants' legal guardians/next of kin in accordance with the national legislation and institutional requirements.

## Author contributions

JW, FL, JY, and JH contributed to conception and design of the study. SS performed the statistical analysis. JW organized the database and wrote the first draft of the manuscript. FG, WD, CC, and FL wrote sections of the manuscript. All authors contributed to manuscript revision, read, and approved the submitted version.
